# Diagnostic value of sialyl‐Tn immunocytochemistry in breast cancer presenting with pathological nipple discharge

**DOI:** 10.1002/cam4.3793

**Published:** 2021-02-19

**Authors:** Feng Xu, Yue Gao, Xiaoli Diao, Jie Li, Hongchuan Jiang, Hongying Zhao

**Affiliations:** ^1^ Department of Breast Surgery Beijing Chao‐Yang Hospital Beijing China; ^2^ Department of General Surgery Beijing Huairou Hospital Beijing China; ^3^ Department of Pathology Beijing Chao‐Yang Hospital Beijing China

**Keywords:** breast cancer, cytopathology, diagnosis, pathological nipple discharge, sialyl‐Tn

## Abstract

**Background:**

Mucin‐associated sialyl‐Tn (sTn) antigen is overexpressed and related with adverse outcome in breast cancer (BC). The role of sTn in BC has not been well defined in pathological nipple discharge (PND) cytology. The authors examined sTn immunocytochemistry (ICC) in PND to determine whether it could be a biomarker of malignancy or aggressive disease.

**Methods:**

PND was subjected to immunocytochemical staining for sTn antigen expression and thinprep cytology test (TCT) for enhancing the sensitivity and specificity. The examination data was compared with histological findings of subsequent biopsy specimens. Logistic regression analysis was used to determine which factors were most associated with malignant breast lesions.

**Results:**

PND specimens were collected including 120 cases of intraductal papilloma, 24 cases of hyperplasia, 45 cases of ductal carcinoma in situ (DCIS), and 48 cases of invasive ductal carcinoma (IDC). STn ICC differentiated BC from benign intraductal lesions with a low sensitivity of 41.9% and a high specificity of 95.8%, but increased in combination with TCT to 64.5% and 100%, respectively. A high degree of concordance was observed between the results of sTn expression in cell smears and histological specimens. Moreover, the sTn expression was strongly associated with HER2‐positive IDC (*p* = 0.039). Multivariate logistic analysis showed that positive sTn expression (OR: 14.241, 95%CI: 2.574, 78.794, *p* = 0.010) and accompanying mass (OR: 3.307, 95%CI: 1.073, 10.188, *p* = 0.037) were statistically significant independent risk factors for malignant PND.

**Conclusions:**

Mucin‐associated sTn expression in PND cytology appears to be a reliable diagnostic marker for BC patients with the chief complaint of malignant nipple discharge and indicates a more aggressive behavior in IDC.

## Graphical abstract

Our study showed that sTn ICC differentiated breast cancer from benign intraductal lesions with low sensitivity of 41.9% and high specificity of 95.8%, but increased in combination with TCT to 64.5% and 100%, respectively. Moreover, sTn expression was strongly associated with HER2‐positive invasive ductal carcinoma. Multivariate logistic analysis further showed that positive sTn expression and accompanying mass were statistically significant independent risk factors for malignant nipple fluid. Therefore, mucin‐associated sTn expression in PND cytology appears to be a reliable and useful marker for early BC detection.

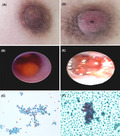

## 1 INTRODUCTION

Globally, breast cancer has become the third highest incident cancer in 2017, with an estimated 1,960,681 incident cases and a high prevalence in females.^1^ Pathological nipple discharge (PND) refers to intermittent and continuous nipple discharge from one or more ducts on one or both sides under non‐physiological conditions. PND is a common chief complaint in patients with breast disease and is indicative of a possible intraductal lesion; thus, it requires further evaluation.^2^ It is most commonly caused by intraductal papilloma (IDP) or benign ductal ectasia but is also associated with underlying malignancies such as ductal carcinoma in situ (DCIS) and invasive ductal carcinoma (IDC). Nipple fluid cytology is widely used for clinical examination. The traditional cytological evaluation by squeezing the nipple is associated with a low diagnostic sensitivity of breast cancer (BC) with the main manifestation of PND. So are the biological markers such as superoxide dismutase (SOD‐1)^3^ and prostate specific antigen (PSA).^4^ Therefore, the identification of a new technique or biomarker capable of detecting the cancer cells in pathological nipple fluid is needed.

As far as we know, thinprep cytology test (TCT) is a high‐quality filming technology in current exfoliated cell examination. TCT examination can improve the detection rates of malignant tumors. This technology is now widely used in cervical cytology examinations and can significantly increase the detection rates of cervical cancer.^5^ The sensitivity of TCT technology in the diagnosis of non‐gynecological cell specimens may be also better than traditional cell‐smear methods. For nipple fluid, more ductal exfoliated cells are collected through duct lavage. The application of TCT may better distinguish benign and malignant breast duct lesions.

Aberrant O‐glycosylation can play a pivotal role in cancer development, progression, angiogenesis and metastasis. These alterations of abnormal glycosylation in cancer cells can result in the exposure of onco‐fetal sialyl‐Tn (sTn) antigen.^6^ STn is a truncated O‐glycan containing a sialic acid‐2,6 linked to GalNAc‐O‐Ser/Thr (Tn antigen), which is associated with an adverse outcome in cancer patients. STn overexpression has been described in many types of epithelial cancer including: pancreatic, ovarian, colorectal, lung, cervical, esophagus, and breast cancer.^7^, ^8^, ^9^, ^10^, ^11^, ^12^

For BC, the expression of sTn has been reported in a few series. Marja Leivonen et al^13^ documented the expression of sTn was associated with larger tumor size and ER/PR negativity. Other published studies have proposed high sTn emerged as an independent prognostic indicator for disease free survival (DFS) and overall survival (OS).^14^ However, the analyses in those studies were carried out on surgical samples. None of those investigators evaluated the diagnostic role of sTn on cytologic specimens that were not fixed in formalin, particularly PND cytology. Therefore, studies validating sTn expression in PND are needed to determine whether sTn can be used as a diagnostic biomarker of breast cancer in cytology. To the best of our knowledge, this is the first study investigating the performance of sTn in nipple fluid cytology.

## 2 METHODS

### 2.1 Subjects

On approval by the Institutional Ethical Committees of Beijing Chao‐Yang Hospital, we evaluated PND specimens from 237 patients who had a chief complaint of PND in our hospital from 2018 to 2020. The detailed inclusion criteria were as follows: (1) histological diagnoses were confirmed using surgical resection or histologic biopsy; (2) each patient gave informed consent before undergoing PND examination; (3) no history of preoperative radiotherapy and chemotherapy; and (4) patients without autoimmune disease and other malignancies.

### 2.2 ThinPrep cytology test

After the application of fiberoptic ductoscopy system (FDS), we used saline to wash and scrap the inductal lesion, and collected PND with a large amount of exfoliated epithelial cells. ThinPrep 2000 processor (Cytyc Company) was used for TCT examination. After centrifuging PND, we placed the cell precipitate in a container with a special buffer fixation solution. Through high‐speed rotation, filtration and other techniques in the ThinPrep 2000 processor, we loosened part of the cell clumps and filtered non‐epithelial components, then transferred these epithelial cells evenly to the slide. Finally, we fixed the slide and made Papanicolaou staining. Cytological findings could be grouped into two categories: cancer cells and non‐cancer cells.

### 2.3 Immunocytochemistry analysis

Nipple fluid in the form of droplets was collected in capillary tubes. Take 200 µl of PND and drop it on the slide. After the cells adhered to the wall, we fixed them with 95% alcohol for 15 minutes, and blocked with 3% hydrogen peroxide. The sections were incubated with anti‐sTn antibody (mouse monoclonal antibody, clone B72.3, dilute: 1:100, Santa Cruz) for one hour at 37°C. The sections were washed with PBS and incubated with horseradish peroxidase‐labeled secondary antibody for 30 min. Subsequently, all sections were visualized with DAB Horseradish Peroxidase Color Development Kit and the nucleus was counterstained with hematoxylin. For positive controls, the samples of sh‐Cosmc MCF‐7 breast cancer cells was used for sTn staining.^15^ Negative controls were prepared by replacing the primary antibody with phosphate buffer saline (PBS). The expression of sTn in resected FFPE tissues was determined through immunohistochemistry (IHC) according to our previous study.^11^

### 2.4 Evaluation of sTn expression

Based on the staining intensity and distribution, semi‐quantitative levels of sTn expression were obtained. Staining intensity (I) was rated as 0 (absent staining), 1 (weak, light yellow), 2 (moderate, yellowish brown), and 3 (strong, brown). The percentage (0%–100%) of reactivity (R) scored as follows: 0 (no positive cells), 1 (positive cells rates <5%), 2 (≥5%), and 3 (≥50%). Histochemistry score = I × R. The cut‐off score of sTn expression less than 2 was classified as negative and the rest as the positive expression. For histologic samples, we used the same staining cut‐off values that were used for the cytologic samples. STn expression was evaluated independently by two different cytopathologists who scored these cytology slides but were blinded to scoring results from the corresponding histology specimens. The average optical density of positive expression in each field of vision was analyzed by the Image‐Pro Plus 6.0 software.

### 2.5 Statistical analysis

Statistical analysis was performed using the SPSS software (version 23.0; IBM). The association between immunostaining markers and clinicopathological variables was explored using either *χ* ^2^ test or Fisher's exact test. Multivariate logistic regression analysis was carried out to identify the clinical and biological parameters likely to predict the presence of breast cancer. In all cases, *p* < 0.05 on both sides was considered as statistically significant.

## 3 RESULTS

### 3.1 Clinicopathological characteristics of the cases

The mean age of the enrolled patients was 35.3 ± 4.8 years. Of 237 enrolled lesions, 93 cases (39.2%) were malignant, and 144 cases (60.8%) were benign. The most common malignancies were invasive ductal carcinoma (IDC; *n* = 48) and ductal carcinoma in situ (DCIS; *n* = 45). Predominant benign lesions were IDP (*n* = 120) and hyperplasia (*n* = 24). Mean lesion size was 10.6 ± 4.4 mm (benign, 8.5 ± 2.7 mm; malignant, 15.2 ± 6.3 mm).

### 3.2 Diagnostic accuracy of TCT and sTn ICC in PND cytology

**FIGURE 1:**
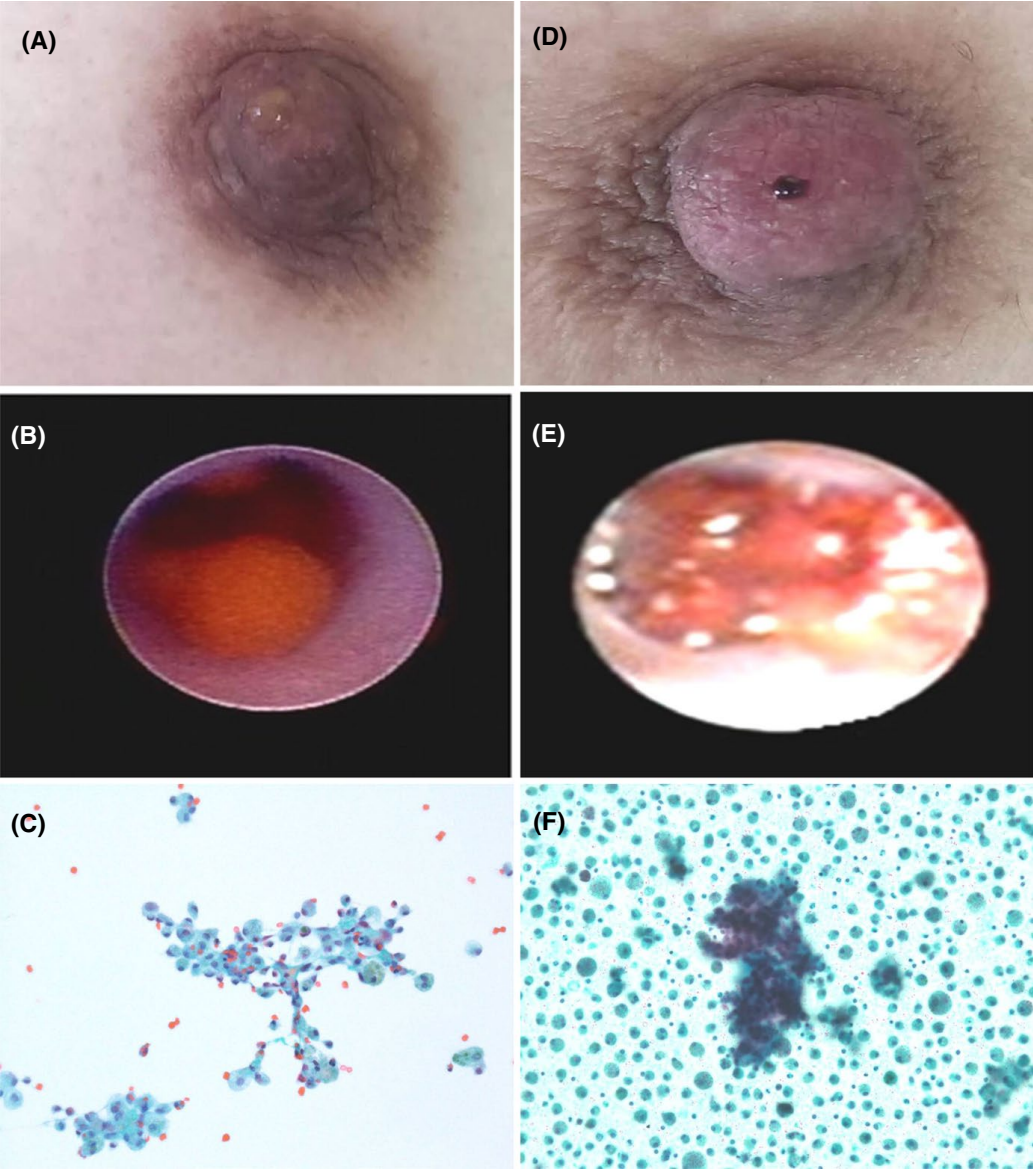
Representative examples of benign (A–C) and malignant (D–F) nipple discharge. (A) PND with light yellow serous liquid; (B) Fiberoptic ductoscopy system showed inductal pappiloma; (C) TCT showed benign ductal cells and foamy macrophages in background of blood (Papanicolaou stain, ×200); (D) PND with spontaneous bloody fluid; (E) Fiberoptic ductoscopy system showed inductal carcinoma; (F) TCT showed focally dyshesive three‐dimensional cell groups of large pleomorphic cells in background of foamy macrophages (Papanicolaou stain, ×200)

The sensitivity and specificity of TCT and sTn ICC for distinguishing BC cases from non‐cancerous cases with benign IDP and hyperplastic ductal epithelial cells were summarized in Table 1 and Figure 1. TCT and sTn ICC were characterized by specificities of 85.4% and 95.8%, respectively. Their combination yielded a sensitivity of 83.9%, which was higher than that of either sTn ICC alone (41.9%) or TCT alone (70.9%). The false negative rate of sTn was 16.1% that is quite high and means that 16.1% of malignant lesions could be missed if sTn test is negative.

**TABLE 1:** Diagnostic usefulness of sTn ICC and TCT in pathological nipple discharge for distinguishing BC from benign lesions

	Malignant *N* = 93		Benign *N* = 144		Sensitivity	Specificity
	Positive	Negative	Positive	Negative		
STn ICC	39	54	6	138	41.9%	95.8%
TCT	66	27	21	123	70.9%	85.4%
STn ICC+TCT	78	15	0	144	83.9%	100%

Abbreviations: ICC, immunocytochemistry; TCT, ThinPrep cytology test.

In addition, we performed sTn IHC staining in 84 cases of corresponding tissue specimens, which were compared with sTn ICC results in PND cytology. Similarly, cut‐off value was also set at two for sTn expression by IHC. Furthermore, it was showed that cytological samples prepared from PND and their corresponding tissue specimens exhibited identical results for sTn IHC.

### 3.3 STn immunostaining in cell smears

**FIGURE 2:**
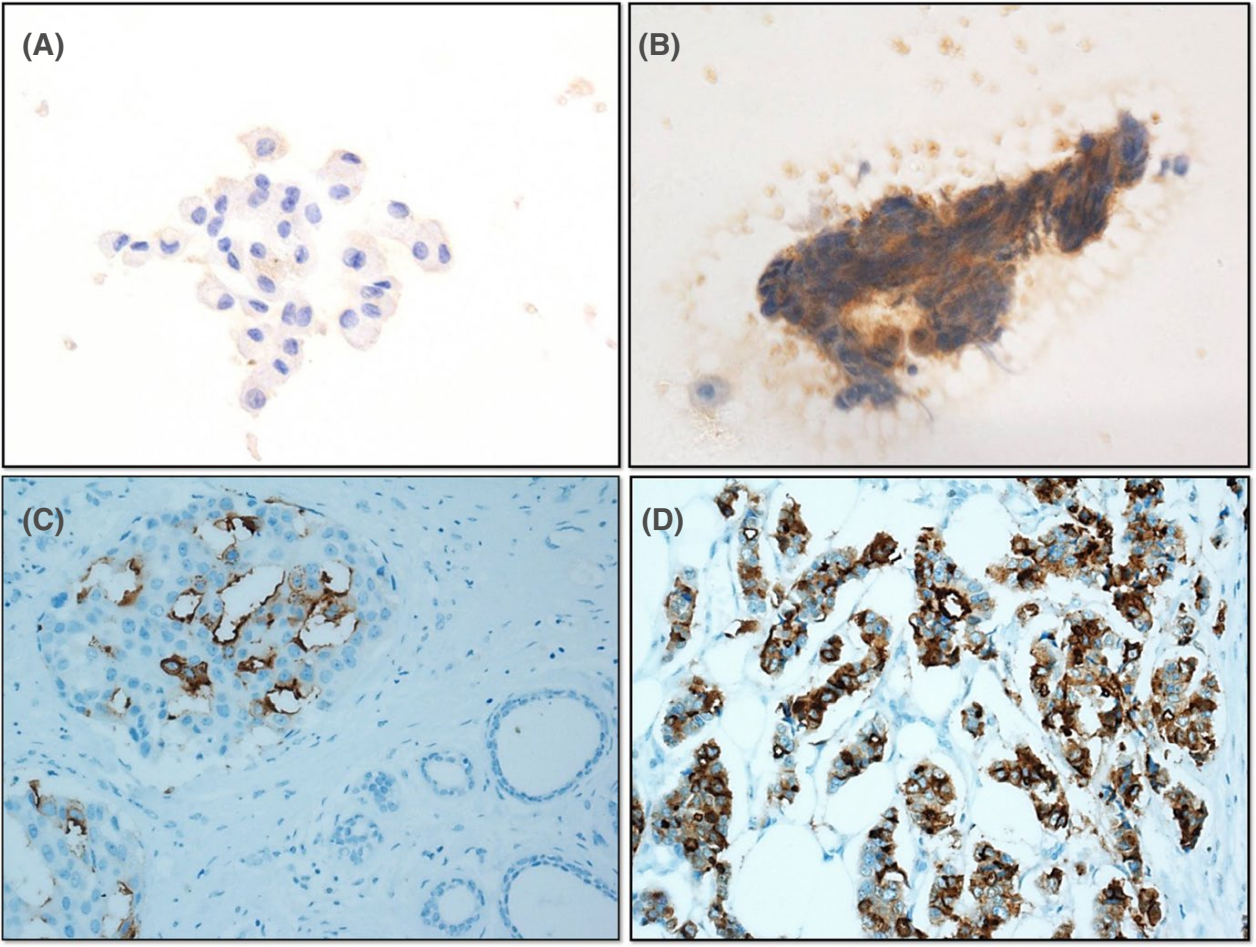
Representative examples of sTn immunostaining in PND cytology and corresponding histological specimens. (A) Negative sTn expression in benign PND cytology with loose ductal cells (×400); (B) Positive sTn expression in malignant PND cytology with discohesive cluster of anaplastic tumor cells (×400); (C) Subsequent negative sTn expression in ductal epithelial cells adjacent to DCIS, which showed apical membrane and cytoplasmic distribution of sTn antigen (×200); (D) Subsequent diffuse‐cytoplasmic positive sTn IHC in coexisting invasive ductal carcinoma (×200)

In our study, sTn antigen was distributed by diffusion and mainly existed in cytoplasm of tumor cells (Figure 2). STn exhibited high expression rates in malignant nipple fluid with values of 41.9% (39/93). The false negative rate is 58.1%, which means that 58.1% of malignant lesions could be missed if the sTn test is negative. The HER‐2 amplification, which we evaluated by fluorescence in situ hybridization or IHC of IDC samples, was correlated with sTn expression in our series (*p* = 0.039, Table 2). Fifteen of 24 cases (62.5%) with sTn expression had positive HER‐2 amplification, indicating a more aggressive behavior in IDC. However, no statistically significant association was observed between the expression of sTn and other clinicopathological features such as tumor size (<2 cm vs. ≥2 cm), tumor type (DCIS vs. IDC), histological Grade of IDC (I vs. II vs. III), nuclear grade of DCIS (low vs. intermediate vs. high), ER and PR status (positive vs. negative) (*p* > 0.05 for all).

**TABLE 2:** Clinicopathological variables associated with sialyl‐Tn expression in malignant nipple fluid cytology

Variable	sTn (+)	sTn (−)	Total	*p* value
Tumor type				
DCIS	21	24	45	0.371
IDC	18	30	48	
Histological grade of IDC				
I	3	6	9	0.927
II	9	12	21	
III	6	12	18	
Nuclear grade of DCIS				
Low	3	6	9	0.192
Intermediate	6	12	18	
High	12	8	18	
HER‐2 on IDC				
Positive	15	9	24	**0.039**
Negative	3	21	24	
ER status on IDC				
Positive	9	12	21	0.696
Negative	9	18	27	
PR status on IDC				
Positive	6	15	21	0.515
Negative	12	15	27	
Tumor size				
<2 cm	30	39	69	0.609
≥2 cm	9	15	24	

sTn(+): positive sTn expression; sTn(‐): negative sTn expression; Total: total number of patients with malignant nipple fluid (bold).

### 3.4 Risk factors affecting malignant nipple fluid

In this study, sTn‐positive cytology accounted for 39 cancer cases, which was significantly different from six non‐cancer cases (*p* < 0.001). The proportions of bloody discharge in benign and malignant breast lesions were 45.8% and 71.0% (*p* = 0.028), respectively. In patients presenting with regular tumor shape the risk of carcinoma was 22.4% (33/147) compared to 66.7% (60/90) in the irregular group (*p* = 0.019). In addition, whether palpable mass was accompanied between benign and malignant nipple discharge were also statistically significant (*p* = 0.010) (Table 3). However, there was no statistically significant difference from the patient's age, breast cancer history, ductal involvement, spontaneous fluid, nipple depression, tumor location and duct obstruction (*p* > 0.05 for all).

**TABLE 3:** Clinical variables and tumor characteristics at presentation of patients with nipple fluid cytology

Variable	Benign	Malignant	Total	*p* value
Age				
<50 years	81	42	123	0.335
≥50 years	63	51	114	
Color				
Bloody	66	66	132	**0.028**
Non‐bloody	78	27	105	
Breast cancer history				
Present	24	15	39	0.950
Absent	120	78	198	
Ductal involvement				
Single	108	60	168	0.317
Multiple	36	33	69	
Accompanying mass				
Present	30	45	75	**0.010**
Absent	114	48	162	
Presentation				
Spontaneous	108	75	183	0.559
Induced	36	18	54	
Nipple depression				
Present	18	18	36	0.407
Absent	126	75	201	
Tumor location				
Duct I	81	33	114	0.177
Duct II	39	33	72	
Duct III	24	27	51	
Tumor shape				
Regular	90	33	123	**0.019**
Irregular	54	60	114	
Blocked duct				
Present	51	45	96	0.252
Absent	93	48	141	
STn expression				
Positive	6	39	45	**<0.001**
Negative	138	54	192	

Benign: benign nipple fluid cytology; Malignant: malignant nipple fluid cytology; Total: total number of patients with nipple fluid cytology (bold).

We further performed a logistic regression analysis (Table 4). The results revealed that only positive sTn expression (OR: 14.241, 95%CI: 2.574, 78.794, *p* = 0.010) and accompanying mass (OR: 3.307, 95%CI: 1.073, 10.188, *p* = 0.037) were statistically significant independent risk factors for malignant nipple fluid.

**TABLE 4:** Multivariate logistic regression analyses of risk factors correlated with malignant breast lesions

Variables	Multivariate analysis		
	OR	95% CI	*p* value
sTn expression Positive/negative	14.241	2.574, 78.794	0.002
Bloody fluid Present/absent	1.942	0.629,5.992	0.248
Tumor shape Irregular/regular	2.483	0.739, 8.347	0.141
Accompanying mass Present/absent	3.307	1.073, 10.188	0.037

Abbreviations: 95% CI, 95% confidence interval; OR, odd ratio.

## 4 DISCUSSION

Sialyl‐Tn, a mucin‐type carbohydrate antigen discovered as a cancer marker in the early 80s, is widely expressed with positive rates ranging from 0% to 70%, and associated with poor prognosis in various cancers.^16^ One mechanism that may result in O‐linked glycoproteins carrying truncated Tn and sTn glycans is the inactivation or lack of expression of Cosmc.^17^ As our recent study showed, Cosmc promoter hypermethylation^18^ was found to decrease the levels of Cosmc and increase the expression of Tn/sTn antigen in breast cancer cells. Senthil R. Kumar reported that the Tn antigen was detected in 92% of the cancerous PND samples through employing competitive inhibition ELISA for quantitation of Tn antigen.^19^ In contrast to Tn, sTn was rarely systematically detected throughout normal adult tissues, meaning that its expression was necessarily pathologic. However, few studies have evaluated the role of sTn in the diagnosis of patients with PND. Here, we have assessed sTn ICC in combination with TCT in both benign and malignant PND specimens.

To the best of our knowledge, the current study is the first to explore the diagnostic value of sTn ICC in PND cytology. Distinguishing cytologically between malignant and benign lesions, including IDC/DCIS and IDP, can be difficult. In the current study, the sensitivity of sTn ICC was relatively unsatisfactory (41.9%), but increased in combination with TCT to 83.9%, which was higher than that of sTn ICC alone. Furthermore, the combination maintained a high specificity of 100%, indicating that it could be used in routine practice for the diagnosis of malignant nipple discharge.

Regarding traditional fluid cytology, the sensitivity and specificity reported in the literature were only 16.7% and 66.1% due to fewer cells by squeezing the nipple.^20^ The TCT examination could collect the effective cell components more comprehensively in nipple discharge, accompanying with evenly distributed cells and clear background. It is reported that the sensitivity of TCT examination is 50%–66.7% for the diagnosis of malignant nipple discharge.^21^ In our study, TCT had a sensitivity of 70.9% and a specificity of 85.4% in diagnosing malignant nipple discharge. The missed cases may be due to the blockage of breast ducts and failure to obtain enough epithelial cells of the distal‐duct lesions. Here, we found that six in fourteen cases were missed by TCT as a result of ductal blockage. In addition, factors such as the extraction and preservation of exfoliated cells during the collection process made it difficult to obtain enough cells for diagnosis.

We further analyzed the relationship between sTn expression and other prognostic parameters of breast cancer. Sores et al. demonstrated that the expression of sTn was higher in breast cancer harboring positive HER‐2 expression.^22^ Similarly, sTn expression was also significantly correlated with HER‐2 amplification evaluated by IHC or FISH of IDC samples in our series, indicating a more aggressive behavior in BC. Twenty‐four cases harbored HER‐2 amplification, including 15 sTn‐positive and 9 sTn‐negative neoplasms. A definitive and significant correlation between sTn and HER‐2, however, has not yet been documented, and the related molecular mechanism should be clarified in future studies.

At present, there is still a lack of objective evaluation criteria in the differentiation of benign and malignant nipple discharge. Rose et al. analyzed 68 patients with PND.^23^ The results showed that the diagnostic sensitivity of rough tumor surface, irregular tumor shape and bloody fluid reached 42.3%, 35.3%, and 45.5%, respectively. The proportions of malignant lesions of bloody discharge in this group of cases were 71.0%, which was higher than those reported in the literature. A retrospective study reported by Makita et al.^24^ showed that more than half of irregularly shaped and multiple intraductal lesions were pathologically diagnosed as malignant breast lesions. In our study, sTn antigen expression in PND cytology, bloody discharge, irregular tumor appearance, and accompanying masses were statistically significant in the diagnosis of BC patients with malignant nipple fluid. Moreover, logistic regression analysis showed that sTn expression in PND and palpable masses accompanied with PND were independent predictors for breast cancer. Therefore, sTn antigen expression might have certain diagnostic value in PND cytology, but its clinical application value still needed to be further improved.

Several limitations involved in this study were underlined as follows. First, the ability of sTn ICC to determine whether a patient with PND had breast cancer was limited by the small number of patients in the study. Second, the current study lacked PND samples with precancerous lesions such as atypical ductal hyperplasia. A study which collected atypical cell samples from women at increased BC risk might help to address the limitation. Finally, we just explored the relationship between sTn and HER‐2 in clinical study. Actually, we should further expand to cell experiments to study the molecular mechanisms of both markers.

In conclusion, we first showed the feasibility of sTn immunostaining in PND, and observed a moderate sensitivity and high specificity in combination with TCT examination. Moreover, a high degree of concordance was observed between the results of sTn expression and positive HER‐2 expression in IDC, indicating the possibility to use the results as an additional prognostic parameter for guiding targeted therapy.

## CONFLICT OF INTEREST

The authors declare that they have no competing interest.

## AUTHORS’ CONTRIBUTIONS

FX, HJ, and HZ conceived and designed the study. FX, YG, XD, and HZ collected the data and performed the experiments. FX and JL performed data analysis. HJ and FX wrote the paper. HZ reviewed and edited the manuscript. All authors read and approved the manuscript.

## Data Availability

All data included in this study are available upon request by contact with the corresponding author.
